# Crystal Structure of Human Nocturnin Catalytic Domain

**DOI:** 10.1038/s41598-018-34615-0

**Published:** 2018-11-02

**Authors:** Michael A. Estrella, Jin Du, Alexei Korennykh

**Affiliations:** Department of Molecular Biology, Princeton, NJ 08544 USA

## Abstract

Nocturnin (NOCT) helps the circadian clock to adjust metabolism according to day and night activity. NOCT is upregulated in early evening and it has been proposed that NOCT serves as a deadenylase for metabolic enzyme mRNAs. We present a 2.7-Å crystal structure of the catalytic domain of human NOCT. Our structure shows that NOCT has a close overall similarity to CCR4 deadenylase family members, PDE12 and CNOT6L, and to a DNA repair enzyme TDP2. All the key catalytic residues present in PDE12, CNOT6L and TDP2 are conserved in NOCT and have the same conformations. However, we observe substantial differences in the surface properties of NOCT, an unexpectedly narrow active site pocket, and conserved structural elements in the vicinity of the catalytic center, which are unique to NOCT and absent in the deadenylases PDE12/CNOT6L. Moreover, we show that in contrast to human PDE12 and CNOT6L, NOCT is completely inactive against poly-A RNA. Our work thus reveals the structure of an intriguing circadian protein and suggests that NOCT has considerable differences from the related deadenylases, which may point to a unique cellular function of this enzyme.

## Introduction

NOCT is a ~50 kDa eukaryotic phosphodiesterase exhibiting an unusual regulation of expression. Nocturnin levels peak in early evening according to the internal circadian clock^1^. In Drosophila, the NOCT gene is called *curled* (curled wing phenotype)^2^. This phenotype is a known signature of a metabolic rather than a developmental defect^2^. Early studies using recombinant protein from *Xenopus laevis* suggested that NOCT can cleave poly-A RNA^3^, leading to the model that NOCT is a deadenylase destabilizing the mRNAs of metabolic enzymes^4^.

NOCT belongs to a multifunctional protein family. Some NOCT homologues are RNA deadenylases^5,6^. However, NOCT is also similar to the DNA repair enzymes such as TDP2^7^ and APE1^3,8^. The closely related deadenylases are CNOT6L^6^ (a mammalian ortholog of the main yeast deadenylase, CCR4) and PDE12 (a mitochondrial deadenylase required for maturation of mitochondrial tRNAs^5^). In contrast to NOCT, none of the related family members are regulated by circadian clock, suggesting that NOCT has a unique and non-redundant function in regulating metabolism. To begin to understand the structural and molecular basis that underlies this cellular function of NOCT, we determined the crystal structure of the catalytic domain from the human protein.

## Results

### Structure of human NOCT reveals catalytic center and fold similar to those of PDE12, CNOT6L, and TDP2

**Figure 1:**
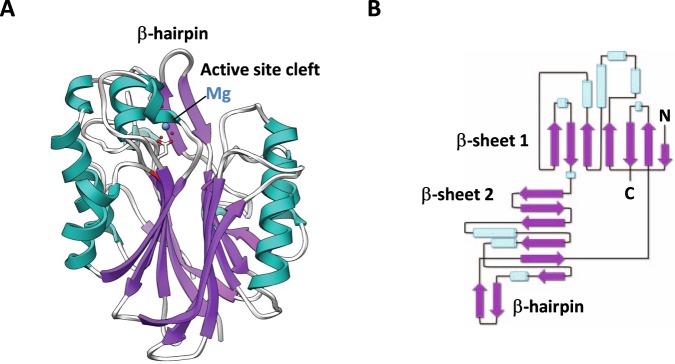
NOCT structure overview. (**A**) Ribbon representation of human NOCT catalytic domain. A single magnesium ion, shown as blue sphere, is located in the catalytic center. (**B**) Topological connectivity diagram of NOCT with secondary structure elements shown.

**Figure 2:**
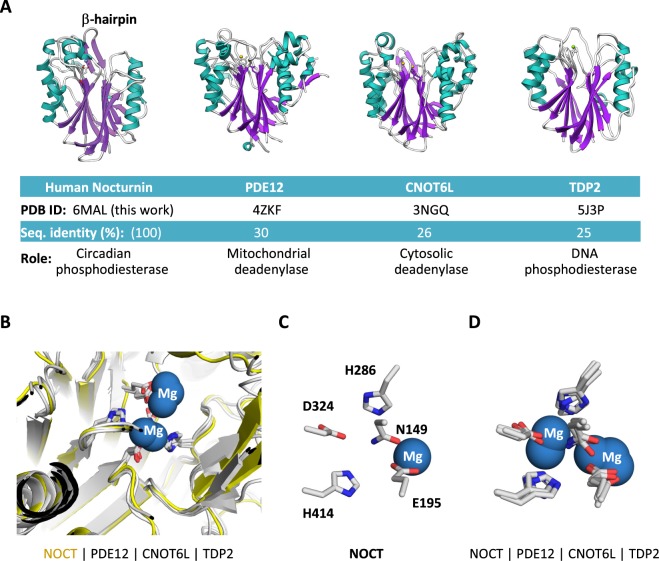
Structural relationships between NOCT and related phosphodiesterases. (**A**) Structures, sequence identity and function summary for NOCT, PDE12, CNOT6L and TDP2. (**B**) Active site superposition for NOCT, PDE12, CNOT6L and TDP2. (**C**) Catalytic residues in the active site of human NOCT. Residues are numbered using human NOCT as a reference. (**D**) Superposition of the catalytic residues in RNA and DNA hydrolases.

We cloned and purified human NOCT and conducted crystallization of both full-length protein and a truncated variant that contains the catalytic domain. The catalytic domain (residues 122–431) produced crystals that diffracted to ~2.7 Å (Table 1). The resulting NOCT structure revealed a globular protein with α/β sandwich fold shared by multiple phosphodiesterases^6^ (Fig. 1A,B). As had been expected based on sequence similarity, the NOCT structure is overall similar to the structures of PDE12 and CNOT6L^3,6^ (Fig. 2A). 3D homology search using DALI server^9^ revealed that NOCT has nearly the same degree of similarity also with TDP2 (Z-score 25.4, RMSD = 2.4 Å), a DNA repair enzyme that resolves topoisomerase stalls by hydrolyzing the phosphodiester link between DNA and tyrosine^7^.

**Table 1:** Data collection and refinement statistics.

	Human Nocturnin, residues 122–431
Data collection	
Space Group	P4_1_2_1_2
Cell dimensions	
a, b, c (Å)	61.4, 61.4, 155.3
α, β, γ (°)	90.0, 90.0, 90.0
Resolution (Å)	30.0–2.7 (2.74–2.69)*
*R*_pim_	0.063 (0.329)
CC(1/2)	0.954 (0.814)
I/σI	10.53 (2.05)
Completeness %	99.9 (99.9)
Redundancy	5.7 (3.8)
Refinement	
Resolution (Å)	2.7
No. unique reflections	9,820 (454)
*R*_work_/*R*_free_	0.2522/0.3069
No. atoms	2,434
Protein	2,396
Mg	1
Water	37
B-factors	
Protein	40.53
Mg	36.13
Water	36.27
R.m.s deviations	
Bond lengths (Å)	0.022
Bond angles (°)	0.7
Ramachandran plot (%)	
Favored	92.7
Allowed	7.0
Disallowed	0.3

^*^Highest-resolution shell is shown in parentheses.

Further extending the analogy with the previously characterized magnesium-dependent phosphodiesterases, NOCT has a magnesium ion bound in the active site (Fig. 2B). All the key catalytic residues that coordinate magnesium and participate in RNA or DNA cleavage by α/β sandwich hydrolases are present in NOCT and occupy the same positions as in the previously described enzymes (Fig. 2C,D). Our structure therefore supports the model that NOCT mediates circadian function by acting as an enzyme hydrolyzing a phosphodiester bond.

### In contrast to human PDE12 and CNOT6L, human NOCT does not cleave poly-A RNA

**Figure 3:**
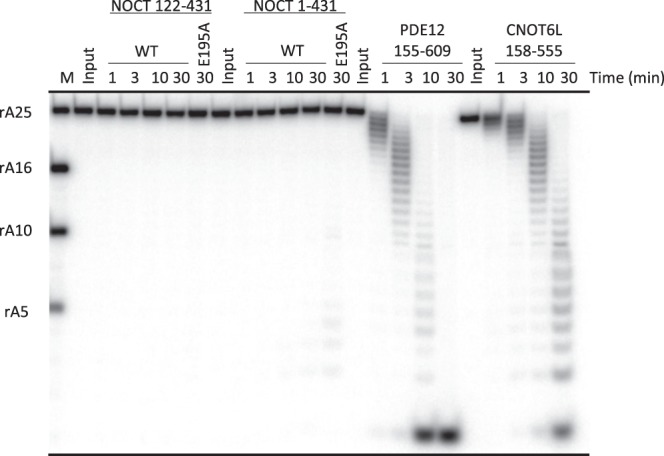
Biochemical analysis of RNA cleavage by NOCT, PDE12 and CNOT6L. 5′-labeled RNA 25-mer (rA25) was incubated with either 2 μM NOCT, 2 μM PDE12, or 2 μM CNOT6L for the indicated times. Four NOCT constructs with residues 122–431 (catalytic domain; WT and E195A catalytic mutant) and 1–431 (full-length; WT and E195A catalytic mutant) were tested. M shows RNA size markers.

To test whether human NOCT is an RNase^3^, we performed poly-A RNA cleavage assays using WT NOCT and NOCT mutant E195A, which removes a conserved residue in the active site. Very unexpectedly, an enzymatic assay revealed a complete absence of poly-A cleavage (Fig. 3). Neither full-length NOCT nor the catalytic domain 122–431 NOCT cleaved poly-A RNA. To track a possible source of this inactivity, we evaluated suitability of our reaction condition for the studies of poly-A RNA hydrolysis. Toward this end, we cloned and purified human CNOT6L (residues 158–555) and human PDE12 (residues 155–609). PDE12 and CNOT6L both readily hydrolyzed poly-A RNA, acting as exoribonucleases as revealed by progressive laddering of the cleave products with time. Therefore, NOCT is functionally different from CNOT6L/PDE12 and does not act as a deadenylase. The unexpected lack of nuclease activity is not consistent with previous reports using murine^10^ and Xenopus^3^ NOCT. However, it agrees with a recent report of human NOCT inactivity published by Abshire *et al*.^11^.

### NOCT has a unique surface character and a surprisingly narrow active site

**Figure 4:**
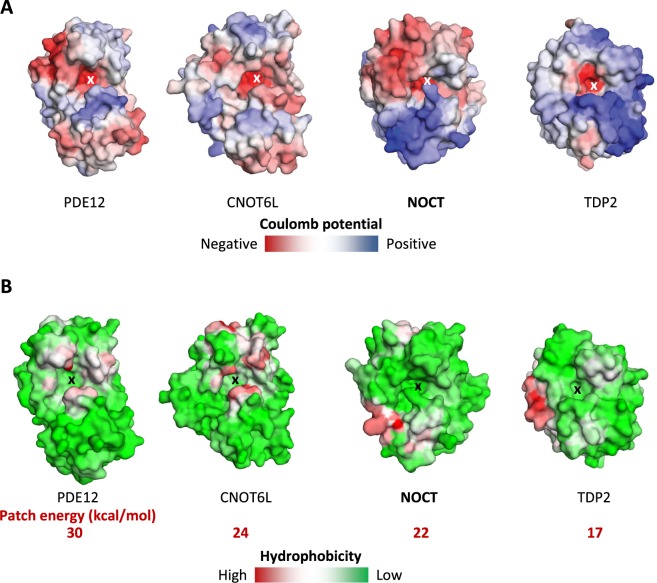
Surface analysis of NOCT and related phosphodiesterases. (**A**) Vacuum electrostatic potential calculated using Coulomb’s law in SEQMOL-Kd (Methods). (**B**) Surface area hydrophobic potential calculated using pre-atom desolvation analysis SEQMOL-Kd. The scale of red color (maximum hydrophobicity) in kcal/mol is shown for each protein.

Although the secondary structure of NOCT is similar to those of PDE12, CNOT6L and TDP2, the surface properties of NOCT have a number of unique features. The global electrostatics of NOCT is distinct from that of the deadenylases PDE12 and CNOT6L due to the presence of a vast acidic area and a vast basic area near the active site in NOCT (Fig. 4A). Neither PDE12 nor CNOT6L have these areas and their electrostatics on the active site face arises largely from the acidic residues in the catalytic center. Although the electrostatic properties of NOCT and TDP2 are different, both proteins have patches of positive charge in similar locations. Surface charge properties of human NOCT are therefore more closely related to TDP2 than to PDE12/CNOT6L (Fig. 4A).

Further differences between NOCT and the canonical deadenylases were revealed upon analysis of the hydrophobic potentials. The deadenylases PDE12/CNOT6L have similar hydrophobic potential configurations, with hydrophobic hot spots flanking the catalytic centers (Fig. 4B). These hot spots likely provide the interaction energy for recognition of nucleobases in poly-A RNA substrates. In contrast, NOCT lacks these hydrophobic hot spots altogether (Fig. 4B). The hydrophobic potential of NOCT resembles that of TDP2 more closely than those of PDE12 and CNOT6L: only NOCT and TDP2 are devoid of the hydrophobic patches around the catalytic core. Moreover, both NOCT and TDP2 evolved a hydrophobic patch on the surface located to the left of the active site (Fig. 4B), which could function as a docking site for partner proteins.

**Figure 5:**
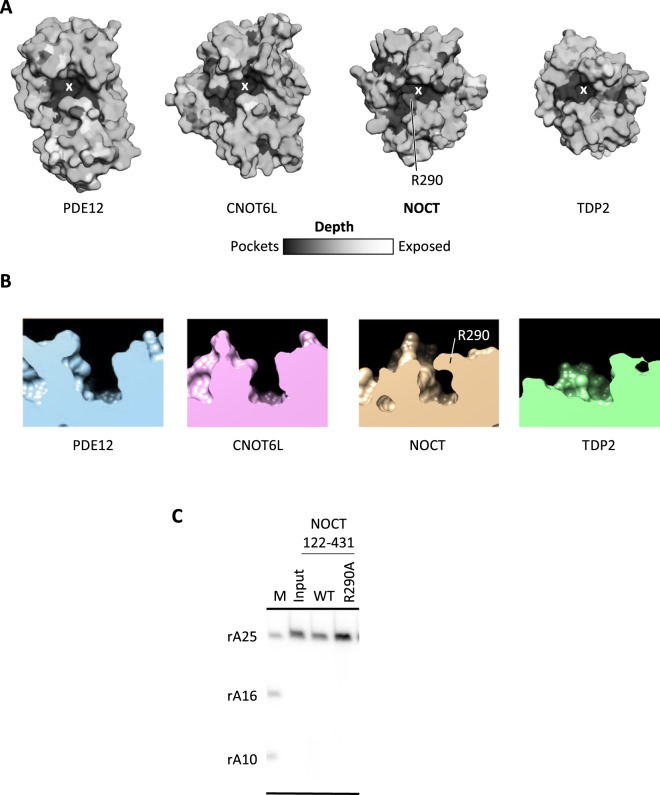
Active site properties of NOCT. (**A**) Accessibility for a probe of 2.5 Å radius calculated in SEQMOL-Kd. Darker areas show deeper pockets. (**B**) Side view (slice) of the active sites in NOCT, PDE12, CNOT6L and TDP2. All proteins were superimposed and positioned identically on the panels. TDP2 has the most open active site, whereas access to the NOCT active site is occluded by R290. (**C**) Recombinantly purified NOCT 122–431 WT and R290A were tested for ribonuclease activity. Reactions were carried out with 20 μM protein at 22 °C for 30 min. “M” lane shows RNA size markers.

Analysis of active site accessibility reveals an additional difference between NOCT and the other α/β sandwich hydrolases. The active site pocket in NOCT is slightly longer than that in the other enzymes (Fig. 5A). Unexpectedly, the active site appears to be closed by a lid created by residue R290 (Fig. 5A,B). The narrow space created by the placement of R290 appears incompatible with binding of RNA, suggesting that R290 may have to move to permit substrate entry. To test this hypothesis, we generated R290A NOCT mutant and performed RNA cleavage assays (Fig. 5C). The mutation of R290A was insufficient for unlocking poly-A cleavage by NOCT, suggesting that other active site differences between NOCT and PDE12/CNOT6L are responsible for the lack of RNA cleavage.

### Conservation analysis reveals unique structural elements near the active site present only in NOCT

**Figure 6:**
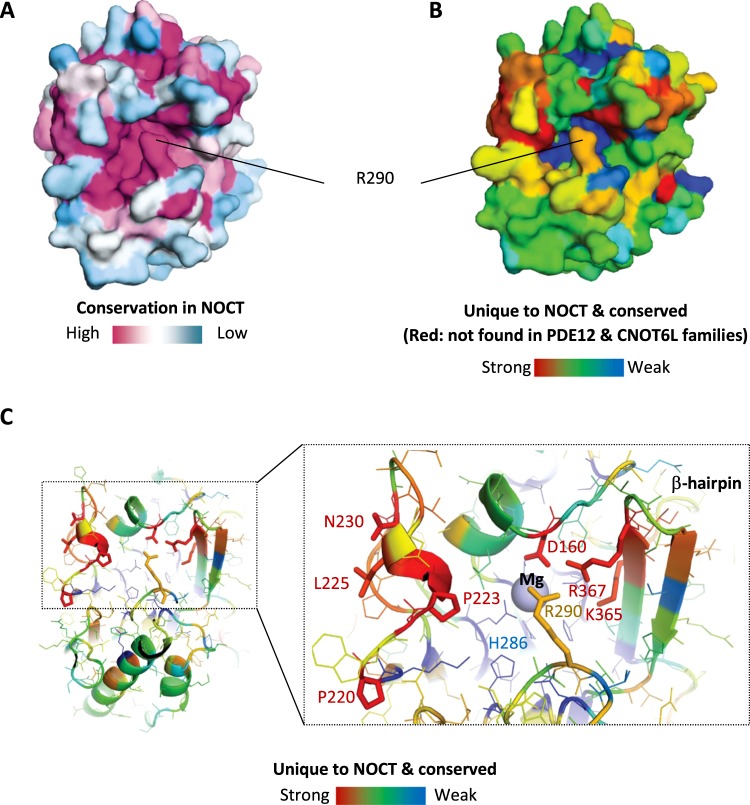
Conservation analysis for NOCT. (**A**) NOCT conservation calculated from 351 non-redundant protein sequences using SEQMOL-Kd. Multiple sequence alignment was conducted with built-in Muscle 3.7^14^. (**B,C)** Modified conservation analysis to visualize residues present only in NOCT family members, but absent in the families of PDE12 and CNOT6L deadenylases. Multiple NOCT-specific residues surround the active site.

Sequence conservation analysis remains one of the most reliable methods to attribute functional importance to protein residues. To obtain the conservation data, we identified 351 non-redundant NOCT sequences^12^ and carried out conservation analysis of NOCT crystal structure using SEQMOL-Kd (Methods). This analysis revealed a strong conservation in the active site of NOCT, including conservation of the lid formed by the residue R290 (Fig. 6A). Therefore, conservation of the catalytic residues and phosphodiesterase activity is important for NOCT function. Moreover, the residue R290 is validated as a conserved part of the NOCT active site that has a yet unknown function.

Considering that NOCT has been conventionally described as a deadenylase^3,4^, we extended the conservation mapping analysis to allow straightforward comparisons of NOCT with PDE12 and CNOT6L. To this end we obtained 339 non-redundant sequences of PDE12 and 785 non-redundant sequences of CNOT6L. We calculated the net conservation scores for all residues in PDE12 and CNOT6L and arranged the scores in register with the NOCT amino acids. Next, for each NOCT residue we calculated the parameter S as follows:

$${\rm{S}}={([\mathrm{NOCT}\_\text{conservation}]\bullet [1-[{\rm{PDE}}12\& {\rm{CNOT}}6{\rm{L}}\_\text{conservation}]])}^{0.5}$$

This modified conservation score (S) will attribute low values to residues conserved in NOCT, PDE12 and CNOT6L, such as catalytic amino acids. The low score will also be attributed to residues that are not conserved in NOCT. High score will be attributed only to NOCT residues that are 1) conserved in the NOCT family and 2) not conserved in the PDE12 and CNOT6L families. The resulting image reveals the structural elements that are invariable in NOCT and unique only in NOCT (Fig. 6B, red). A close-up view of the S-colored PDB structure shows that in addition to the residue R290, NOCT harbors a number of other highly conserved NOCT-specific residues around the active site. These residues include P220, P223, L225 and N230 in the coil to the left of the active site, D160 in the alpha-helix-loop motif above the active site, and K365 and R367 in the β-hairpin (Fig. 6C).

## Discussion

Our structure of human NOCT reveals the expected resemblance with the deadenylases PDE12 and CNOT6L and also a similarity with the DNA repair enzyme TDP2. The electrostatic properties and especially the hydrophobic properties of NOCT are nevertheless considerably divergent and unique. We found that NOCT does not have the hydrophobic spots surrounding the catalytic pockets in both PDE12 and CNOT6L, serving as nucleobase docking sites for poly-A. We do not fully understand how NOCT recognizes RNA without these hydrophobic areas. However, the absence of these nucleobase binding hydrophobic surfaces in NOCT could be the reason for the inability of NOCT to cleave RNA in our work and in the work of Abshire *et al*. (Note added in revision).

We found further that the active site of NOCT is occluded by the residue R290. Eliminating this residue via R290A mutation did not unlock the poly-A cleavage activity of NOCT (Fig. 5C), suggesting that NOCT is inactive for more than one reason. The residue R290 is likely to participate in substrate recognition by NOCT and is likely to move to allow substrate entry. Whereas side chains often can move and R290 could potentially change conformation upon substrate docking, conformational changes have energetic costs. The required free energy will inevitably weaken the affinity for RNA. However, conformational changes can provide the advantage because they can act as switches regulating the catalytic activity. The evolutionarily importance of R290 and the expectation that R290 has to move to allow RNA entry suggest that NOCT could be a regulated, rather than a constitutively active enzyme.

Our efforts to co-crystallize NOCT with poly-A and poly-dA, including tests with NOCT catalytic mutants, produced only apo crystals. These results are in line with the inability of NOCT to cleave poly-A (Fig. 3; Note added in revision). Interference with R290 could be one of the explanations for the difficulty of capturing NOCT with nucleic acids bound. Alternatively, it is possible that NOCT is a pseudoenzyme. It is also possible that NOCT cleaves RNA other than poly-A, or cleaves either DNA or another cellular target. Further understanding of NOCT must await the availability of a structure between NOCT and its cognate substrate.

## Materials and Methods

### Cloning

The coding region of full-length human NOCT(1–431) was amplified by PCR from an in-house cDNA library using polyI:C transfected A549 cells and cloned into pGEX-6P vector (GE Healthcare Life Sciences). The NOCT 122–431 construct was made using site-directed mutagenesis that deleted DNA base pairs corresponding to residues 1–121. The constructs for human PDE12 155–609 and CNOT6L 158–555 were similarly cloned into pGEX-6P, which contains an N-terminus GST fusion tag. E195A and R290A mutants for NOCT 1–431 and 122–431 were generated via site-directed mutagenesis. All constructs used in this study were verified by DNA sequencing.

### Protein purification

pGEX-6P vector containing full-length human NOCT was transformed into *Escherichia coli* BL21 (DE3)-CodonPlus RIPL (Agilent Technologies) and grown to an OD600 of 0.4 in Luria-Bertani medium at 37 ^o^C followed by induction with 0.2 mM isopropyl-β-D-thiogalactopyranoside (IPTG) and overnight expression at 22 ^o^C. The cells were pelleted at 4,600 × g for 20 min, resuspended in lysis buffer [20 mM HEPES (pH 7.4), 1 M KCl, 1 mM EDTA, 10% (vol/vol) glycerol, 5 mM DTT, and 1 × Roche cOmplete protease inhibitors], and lysed on an EmulsiFlex C3 (Avestin). Crude lysates were clarified by centrifugation at 35,000 × g for 30 min, at 4 °C. Clarified lysates were affinity-purified using glutathione Sepharose (GE Healthcare Life Sciences) and the GST tag was removed with Prescission protease (GE Healthcare Life Sciences). NOCT 122–431, PDE12 155–609 and CNOT6L 158–555 were similarly purified to this point. Full-length NOCT was further purified by MonoQ, then MonoS ion-exchange chromatography, and lastly by Superdex 200 size-exclusion chromatography. NOCT 122–431 was further purified using a slight variation of performing MonoS first, then MonoQ ion-exchange chromatography, followed by Superdex 200 size-exclusion chromatography. Purification of NOCT 1-431 and 122–431 mutants, E195A and R290A, were processed exactly as wild-type. For both PDE12 155–609 and CNOT6L 158–555, size-exclusion chromatography was sufficient to yield highly purified protein. Size-exclusion buffer used for all protein constructs consisted of 20 mM HEPES (pH 7.4), 350 mM KCl, 1 mM EDTA, 10% (vol/vol) glycerol, and 5 mM DTT. All proteins were purified to more than 95% purity and concentrations were quantified by UV spectrophotometry.

### Preparation of labeled oligonucleotides

RNA oligonucleotides were purchased from Integrated DNA Technologies. 2 pmol of nucleic acid were 5′ radiolabeled with T4 polynucleotide kinase (New England BioLabs) and γ−32P ATP (Perkin Elmer) in 1 × T4 polynucleotide kinase buffer for 30 min at 37 °C. The substrates were resolved on a denaturing polyacrylamide gel, visualized by autoradiography, excised from gel, and placed in a 0.3-mL solution of 0.3 M sodium acetate overnight at 4 °C followed by ethanol precipitation and resuspension in sterile water.

### RNA cleavage assays

Kinetics analyses with radiolabeled nucleic acid substrates were carried out at 22 °C using the concentrations of 2 nM nucleic acid and 2 μM enzyme, unless otherwise indicated. Reactions contained 5 mM HEPES pH 7.5, 70 mM KCl, 2 mM MgCl_2_, 5% (vol/vol) glycerol, 1 mM DTT, 150 μM Spermidine, and 0.02% NP-40, as used in Baggs *et al*.^3^. The reactions were stopped at indicated times with the addition of quencher dye (90% formamide, 2.5% glycerol, 0.01% SDS, 0.01% bromophenol blue, 0.01% xylene cyanol, 1 mM EDTA) and heated for 10 min at 95 °C. The samples were then run on 20% polyacrylamide denaturing gels and visualized by phosphorimaging.

### Nocturnin 122–431 crystallization

Crystallization drops of NOCT 122–431 contained 17 mg/mL of protein and crystals were grown using the hanging drop vapor diffusion method by mixing the crystallization complex 1:1 with reservoir solution (0.1 M MgCl_2_, 0.1 M HEPES-Na pH 7.5, 30% PEG 400). Crystals were directly frozen in liquid nitrogen.

### X-Ray data collection and structure determination

X-ray diffraction data were collected using our Core facility Rigaku MicroMax-007 HF rotating anode generator supplied with Pilatus3 R 300 K hybrid pixel array detector. Data were collected at a wavelength of 1.54 Å. The crystals were large and well formed and diffraction looked usual, however the data could not be indexed and integrated in HKL2000 and required XDS package for processing. Crystals contain one NOCT 122–431 molecule in the asymmetric unit and belong to the tetragonal P4_1_2_1_2 space group. The structure was solved by molecular replacement in PHASER using human CNOT6L (PDB ID code 3NGO) as the search model. The structure was modeled in COOT and refined by simulated annealing using PHENIX.

### Structure visualization and analysis

Structures were visualized with PyMol (DeLano Scientific build) and UCSF Chimera^13^. Surface properties (conservation, hydrophobic potential, electrostatics, pocket accessibility) were calculated and mapped using SEQMOL-Kd (BiochemLabSolutions, http://biochemlabsolutions.com/FASTAandPDB.html).

### Database entries

Structure factors and coordinates were deposited to PDB database (RCSB.org) under accession ID 6MAL.

### Note added in revision

After our manuscript was completed and deposited to BioRxiv, a study by Abshire *et al*.^11^ reported an independently determined crystal structure of human NOCT. RMSD between our structure 6MAL and the independently published structure 6BT1 is 0.895 Å (all atom) and 0.563 Å (CA). This publication is complementary to our work. It presents a similar structure and comes to the same conclusion that NOCT does not cleave poly-A RNA.
